# Evaluating the Efficacy of Perfusion MRI and Conventional MRI in Distinguishing Recurrent Cerebral Metastasis from Brain Radiation Necrosis

**DOI:** 10.3390/brainsci14040321

**Published:** 2024-03-27

**Authors:** Anders Schack, Jan Saip Aunan-Diop, Frederik A. Gerhardt, Christian Bonde Pedersen, Bo Halle, Mikkel S. Kofoed, Ljubo Markovic, Martin Wirenfeldt, Frantz Rom Poulsen

**Affiliations:** 1 Department of Neurosurgery, Odense University Hospital, DK-5000 Odense, Denmark; 2 Department of Clinical Research, BRIDGE (Brain Research—Inter Disciplinary Guided Excellence), University of Southern Denmark, DK-5230 Odense, Denmark; 3 Department of Radiology, Odense University Hospital, DK-5000 Odense, Denmark; 4 Department of Pathology, University Hospital of Southern Denmark, DK-6000 Esbjerg, Denmark; 5 Department of Regional Health Research, BRIDGE (Brain Research—Inter Disciplinary Guided Excellence), University of Southern Denmark, DK-5230 Odense, Denmark

**Keywords:** cerebral metastasis, radiation necrosis, radiation therapy, magnetic resonance imaging, diagnostic reliability

## Abstract

Differentiating recurrent cerebral metastasis (CM) from brain radiation necrosis (BRN) is pivotal for guiding appropriate treatment and prognostication. Despite advances in imaging techniques, however, accurately distinguishing these conditions non-invasively is still challenging. This single-center retrospective study reviewed 32 cases (28 patients) with confirmed cerebral metastases who underwent surgical excision of lesions initially diagnosed by MRI and/or MR perfusion scans from 1 January 2015 to 30 September 2020. Diagnostic accuracy was assessed by comparing imaging findings with postoperative histopathology. Conventional MRI accurately identified recurrent CM in 75% of cases. MR perfusion scans showed significantly higher mean maximum relative cerebral blood volume (max. rCBV) in metastasis cases, indicating its potential as a discriminative biomarker. No single imaging modality could definitively distinguish CM from BRN. Survival analysis revealed gender as the only significant factor affecting overall survival, with no significant survival difference observed between patients with CM and BRN after controlling for confounding factors. This study underscores the limitations of both conventional MRI and MR perfusion scans in differentiating recurrent CM from BRN. Histopathological examination remains essential for accurate diagnosis. Further research is needed to improve the reliability of non-invasive imaging and to guide the management of patients with these post-radiation events.

## 1. Introduction

Cerebral metastasis (CM) is a prevalent and serious oncological manifestation that develops in an estimated 20% of patients with cancer. Lung, breast, and melanoma cancers are the primary malignancies that most frequently spread to the brain. Modern advances in diagnostic imaging and systemic oncological therapies have enhanced our ability to detect CM. This is a reflection not only of improved diagnostic capabilities but also of extended patient survival, leading to a higher incidence of brain metastases being identified over the prolonged course of the disease. Paradoxically, this increases the challenge of differentiating CM from brain radiation necrosis (BRN) [1,2].

The management of CM encompasses a range of treatments, from surgical resection with adjunctive radiotherapy (RT) to various radiotherapeutic strategies, including whole-brain radiotherapy (WBRT). Stereotactic radiosurgery (SRS) has gained traction as a preferred treatment, boasting high rates of one-year tumor control and fewer adverse effects relative to WBRT [3,4,5].

However, the extended survival of cancer patients and the intensified use of radiotherapy have led to a rise in late-stage radiation complications like brain radiation necrosis (BRN), with an incidence rate of 5–25% post-SRS [6]. This phenomenon, which mimics tumor growth on imaging, actually signifies a benign, post-treatment inflammatory response rather than true disease progression. Another term for this is pseudo-progression. This presents a significant clinical challenge due to its varying clinical presentation and the critical need for its early identification and management. The clinical presentation of BRN can span from asymptomatic changes observed on radiological imaging to debilitating neurological decline, necessitating its early identification and management. The therapeutic approach to BRN is variable and can involve observational strategies, medical management with corticosteroids and VEGF-inhibitors, or surgical intervention in cases of significant mass effect. Conversely, recurrent CM typically demands aggressive treatment aimed at potential cures such as surgery, repeat SRS, WBRT, and chemotherapy [7]. Distinguishing between CM and BRN is crucial in clinical neuro-oncology, as it significantly influences treatment decisions and patient outcomes.

Accurately differentiating between recurrent CM and BRN using imaging techniques is crucial due to their distinct management pathways. While conventional magnetic resonance imaging (MRI) has limited positive predictive value [8], advanced functional imaging techniques like MR perfusion imaging, which evaluates cerebral hemodynamic variations, are emerging as potential alternatives. The rationale behind this is the high microvascular density in CM compared to BRN. Perfusion imaging may be sensitive to this difference.

Two main perfusion MRI techniques exist. The first technique includes T2*-based dynamic susceptibility contrast (DSC) and T1-based dynamic contrast-enhanced (DCE*) MRI. The second technique is called arterial spin-labeling (ASL). A meta-analysis from 2016 indicated that the mean rCBV in a contrast-enhancing lesion was significantly higher in CM compared with BRN (*p* = 0.001) [9]. The analysis included data from ten MR perfusion studies and reported publication bias as a possible limitation. A systematic review and meta-analysis from 2019 reported inconclusive results [10]. While MR perfusion imaging shows promise in evaluating cerebral hemodynamic variations, the clinical and diagnostic utility of perfusion-based methods in the evaluation of CM remain a topic of debate; thus, further investigations are warranted.

A promising area of research lies in the application of quantitative imaging analysis tools, such as machine learning algorithms, to perfusion imaging data. These advanced computational methods have the potential to uncover subtle, yet clinically significant, differences in the perfusion patterns of CM and BRN. For instance, machine learning can analyze the complex interplay of perfusion parameters, peak height, and recovery signals in a more nuanced manner than conventional radiological interpretation.

In addition, integrating MR perfusion data with other imaging modalities, such as MR spectroscopy or PET scans, could lead to more comprehensive diagnostic profiles. This multimodal approach might enhance the specificity and sensitivity of diagnosing CM and BRN, potentially reducing the rate of misdiagnosis and incorrect treatment planning.

MR perfusion imaging is increasingly utilized to distinguish between pseudo-progression and true tumor progression. By measuring blood flow and volume in brain tissues, MR perfusion helps to identify the different vascular characteristics of tumor growth compared to treatment-induced changes. For example, Brandes et al. (2008) [11] demonstrated the utility of MR perfusion in distinguishing pseudo-progression from genuine tumor growth by evaluating changes in relative cerebral blood volume (rCBV). Lesions exhibiting pseudo-progression typically show distinct perfusion patterns, often characterized by lower rCBV, in contrast to the increased vascular activity seen in actual tumor progression.

Our retrospective study directly addresses a key gap in neuro-oncology: the need for more effective differentiation between recurrent CM and BRN using MRI techniques. We specifically examine the extent to which conventional contrast-enhanced MRI and MR perfusion imaging align with histopathological outcomes, a crucial factor for accurate diagnosis and effective patient treatment. Our central hypothesis is that enhanced precision in these imaging modalities can significantly influence patient survival outcomes. By determining the accuracy of these diagnostic tools in identifying cerebral lesions post-treatment, our study aims to provide insights that could lead to better-targeted therapies and improve patient care in neuro-oncology.

## 2. Materials and Methods

### 2.1. Study Design

This was a retrospective cohort analysis conducted at a single center. This study was approved by the Danish National Committee on Health Research Ethics in the Region of Southern Denmark (ID: 20/39028) and the Danish Data Protection Agency (ID: 21/15275). As per Danish health legislation, informed patient consent was not required as this was a retrospective cohort study that had been approved at a regional level. This study was conducted in accordance with the Declaration of Helsinki and adhered to the STROBE (Strengthening the Reporting of Observational Studies in Epidemiology) guidelines.

### 2.2. Patient Selection

Potential study participants were identified from medical records as individuals who met the following criteria:

(i) Received conventional contrast-enhanced cerebral MRI scan and/or cerebral MR perfusion imaging in the Department of Neurosurgery, Odense University Hospital, between 1 January 2015 and 30 September 2020.
(ii) Had a history of intracranial metastatic cancer, i.e., diagnosed with both ICD10 code C793 (secondary malignant neoplasm of brain and meninges) and A+ZCA4 (earlier reported case of malignancy).
(iii) Previous cranial radiation therapy for cerebral metastasis (either as primary treatment or adjuvant therapy).
(iv) Had a lesion suspected to be recurrent cerebral metastasis based on conventional contrast-enhanced cerebral MRI scan and/or cerebral MR perfusion imaging during the period specified above.

All included patients had undergone surgical resection of the lesion during the defined period and had the diagnosis confirmed by histopathological analysis. Radiotherapy in the form of isolated WBRT, stereotactic radiosurgery, or a combination of these was accepted.

All surgeries were performed by a dedicated team of oncological neurosurgeons. Histopathological analysis was performed by a specialist in neuropathology. BRN was defined as either scar tissue, necrotic tissue and signs of inflammation (hyalinization/necrosis of small arteries, perivascular lymphocyte infiltration, etc.), or a combination of these, without any viable tumor cells.

### 2.3. Data Acquisition

Patient data were obtained from electronic patient journals (Cosmic, Cambrio, 8200 Aarhus N, Denmark) in the Department of Neurosurgery. Data were collected on radiotherapy and cerebral surgical interventions, the modality of pathological scan and a description of its findings, the histopathological diagnosis, demographics, relevant clinical characteristics, and survival status up until 4 January 2021. Data were stored and managed through a secure web-based platform (Microsoft Sharepoint).

The primary cancer diagnosis was grouped according to anatomical location and histology. Patient preoperative overall health and performance status was assessed using the American Society of Anesthesiologists (ASA) score, ranging from 1 to 6 [12]. The primary outcome was defined as the result of the histopathological analysis and was dichotomized as either BRN or recurrent CM.

### 2.4. Imaging Procedures

MR imaging was performed in the Department of Radiology, Odense University Hospital, using two 3.0T clinical MR scanners (Achieva or Ingenia Elition, Philips, Amsterdam, the Netherlands) with 15-element Sense Head Coils. A paramagnetic gadolinium (Gd3+)-based intravascular contrast agent (Gadovist^®^ Bayer, Leverkusen, Germany) was administered intravenously as a bolus (0.1 mL/kg, 1.0 mmol/mL, injection rate: 2 mL/s) and this was followed by a 20 mL saline flush. All scans were performed using a preset soft-contrast window, and the patient was placed head-first in a supine position.

#### 2.4.1. Contrast-Enhanced Cerebral MR Imaging

Conventional contrast-enhanced MRI pulse sequences included either axial T1-weighted, T2-weighted, or fluid-attenuated inversion recovery (FLAIR).

T1-weighted scan: FFE/TFE (Fast/Turbo Field Echo) gradient sequence; TR/TE: 7.9/3.5 ms; flip angle: 8°; field of view (FOV): 240 × 200 × 143.1 mm; matrix: 240 × 200; 159 transverse slices; total scan duration: 03:31.7.

T2-weighted scan: TSE (Turbo Spin Echo) sequence; TR/TE: 4000/104 ms; flip angle: 90°; FOV: 240 × 200 × 143 mm; matrix: 400 × 400; 24 transverse slices of 5 mm thickness with a 1 mm gap; total scan duration: 02:31.0.

FLAIR: TSE (Turbo Spin Echo) sequence; TR/TE: 4800/307 ms; flip angle: 40°; FOV: 240 × 200 × 142.8 mm; matrix: 216 × 177; 255 transverse slices; total scan duration: 04:42.0. The inversion time (TI) for the FLAIR sequence was 1650 ms.

As is standard, contrast enhancement was used to indicate a disruption of the blood-brain barrier, which is a typical malignant feature of neo-angiogenesis and is characterized by increased and abnormal vascular organization [10,13,14]. We assessed the localization and intensity of enhancement as well as the degree of vasogenic edema and mass effect.

#### 2.4.2. DSC MR Perfusion Imaging

Dynamic susceptibility-weighted contrast-enhanced (DSC) perfusion images were obtained using a T2*-weighted gradient-echo echo-planar (GRE-EPI) sequence. TR/TE: 1606/40 ms; flip angle: 75°; FOV: 224 × 224 × 100 mm; matrix: 96 × 94; 25 transverse slices of 4 mm thickness with a 0 mm gap; and total scan time: 01:09.1.

We quantitatively assessed hemodynamic variables of cerebral blood flow (CBF), cerebral blood volume (CBV), mean transit time (MTT), and relative cerebral blood volume (rCBV). Lesional rCBV was determined by comparison with the contralateral Gd3+-enhanced hemodynamic pattern of non-pathological gray/white matter. Between three and five regions of interest (ROIs) were defined for each lesion. Correspondingly, an equal number of ROIs were designated on a contralateral site. A relative cerebral blood volume (rCBV) threshold of 1.75 was used to classify a lesion as either recurrent CM or BRN.

All images were evaluated by a single neuroradiologist. The radiologist was effectively blinded to the diagnosis, as histopathological diagnosis was first available after surgery.

### 2.5. Statistical Analysis

Categorical data, including histopathological diagnosis and mortality, were reported as a proportion of the total count of events. Demographic data and clinical characteristics were analyzed and presented as descriptive data. Numerical data were presented as median and interquartile range.

Possible confounding covariates such as gender and radiation exposure were accounted for by stratified analysis for significance. We used the Mann–Whitney U test for significance testing of numerical data and Pearson’s chi-squared test to compare categorical variables with more than two groups of dependent variables. Fisher’s exact test was used to compare the dichotomous variables concerning the primary outcome. Significance testing between two proportions used z-tests.

Survival analysis was performed using the Cox proportional hazards model and Kaplan–Meier estimation of survival rate. These were adjusted for age, gender, radiation exposure, type of scan, and preoperative ASA score. Kaplan–Meier curves of estimated survival rate were produced for both BRN and CM using unadjusted and adjusted estimates. The two adjusted curves were compared using a stratified log-rank test for equality. We conducted an additional survival analysis where the WBRT group was excluded and compared the stratified group with the log-rank test. Multivariate Kaplan–Meier curves were obtained using a Cox proportional hazards model. This model adjusted for significant covariates like age, gender, and type of radiation therapy. From this, we calculated individual predicted survival probabilities and created a stratified survival function. These steps allowed us to plot Kaplan–Meier curves that reflected adjusted survival experiences, accounting for multiple covariates.

Statistical analysis was performed using STATA 16.0 (StataCorp LLC, College Station, TX, USA). *p* < 0.05 was chosen as the threshold for statistical significance.

For the statistical analysis of radiological parametric data, rCBV and rCBF were computed by dividing the CBV (or CBF) of the lesion by that of the side that appeared normally. Both maximum and minimum values were extracted. Statistical analysis was conducted using Python 3.9.13. Fisher’s exact test was used to evaluate the correlation between categorical histopathological and radiological diagnoses. A one-sided *t*-test was applied on the assumption that the max. rCBV and max. rCBF values were higher in recurrent metastases compared to radiation necrosis.

## 3. Results

**Figure 1:**
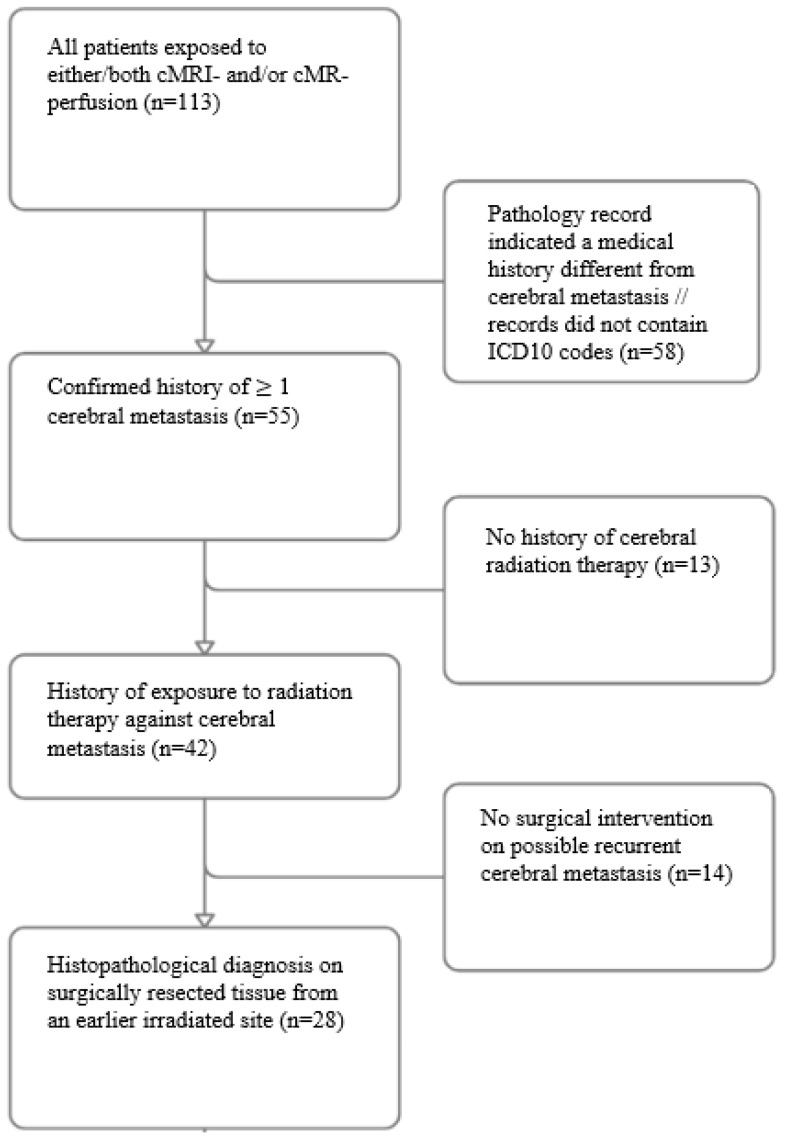
Flowchart illustrating patient inclusion/exclusion. cMRI-scan/cMR-perfusion: cerebral MRI-scan/cerebral MR-perfusion.

From an initial cohort of 113 cases, 85 were excluded due to the absence of a confirmed cerebral metastatic history or lack of prior radiation therapy or surgery, narrowing the study group to 28 patients (Figure 1). Of these, 20 underwent conventional MRI, 4 underwent MR perfusion, and 4 underwent both imaging modalities; this gave 24 conventional MRI and 8 MR perfusion analyses. No significant differences in demographic or clinical confounders were found between the imaging groups. Univariate stratified analysis thus showed no significant differences between the two groups in terms of age (*p* = 0.597), gender (*p* = 0.776), primary cancer (*p* = 0.754), radiation exposure (*p* = 0.147), preoperative ASA score (*p* = 0.926), mortality (*p* = 0.060), or months from radiation to surgery (*p* = 0.913) (Table 1).

### 3.1. Diagnostic Accuracy

In the 24 cases where conventional MRI scan indicated recurrent CM, 18 cases (75%) were confirmed as recurrent CM by histopathological diagnosis, while the remaining 6 cases (25%) were diagnosed as BRN.

**Figure 2:**
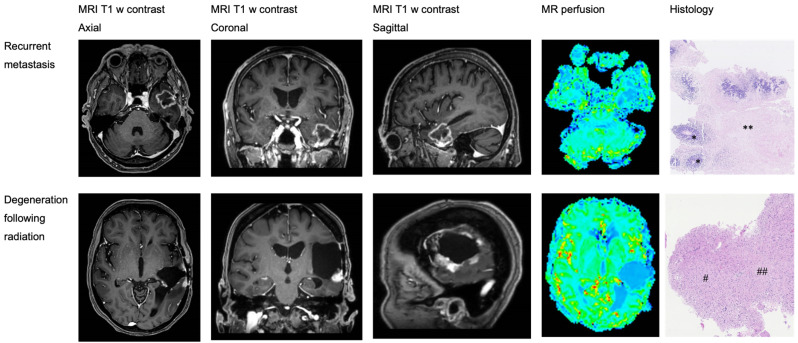
Radiological and histological presentation of cerebral metastasis versus brain radiation necrosis following radiation therapy. Scale bar of 0–100 µm/1 mm. for histological presentation. Upper: cerebral tumor tissue from the temporal lobe with metastatic adenocarcinoma (*) and large necrotic areas (**). The colors in the figure represent variations in cerebral blood flow as detected by MR perfusion imaging. Lower: cerebral tissue from the temporoparietal lobe with gliotic (#) and degenerative (##) changes following radiotherapy.

Table 2 presents a comparison of diagnostic accuracy and imaging parameters of MR perfusion scans. All four patients (100%) with radiation necrosis had a consistent radiological diagnosis, while three out of four patients (75%) with metastasis had a radiological diagnosis that was consistent with the histological diagnosis. The difference between the accuracy of different histological diagnoses was not statistically significant (*p* = 0.14). The mean maximum relative cerebral blood volume was significantly higher in the metastasis group compared to the radiation necrosis group (*p* = 0.048), while the mean maximum relative cerebral blood flow was non-significantly higher in the metastasis group (*p* = 0.051). Data for individual patients are shown in Supplementary Table S1. Figure 2 shows radiological and histological presentation of cerebral metastasis versus brain radiation.

### 3.2. Survival

**Figure 3:**
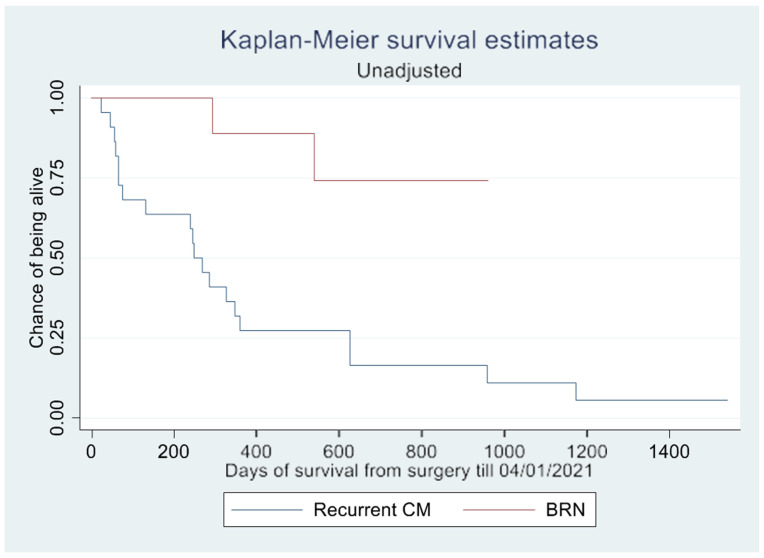
Unadjusted Kaplan–Meier survival estimates. Abbreviations: CM, cerebral metastasis; BRN, brain radiation necrosis.

**Figure 4:**
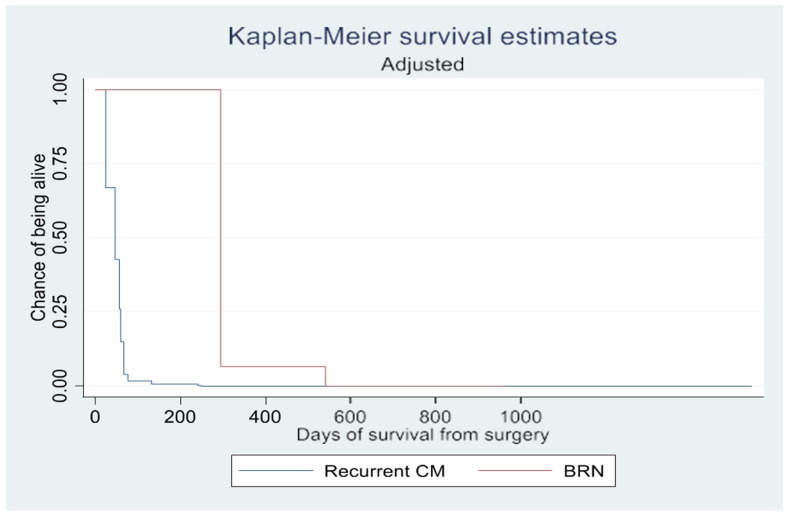
Kaplan–Meier survival estimates adjusted for type of scan, gender, age, radiation exposure, ASA score, and primary cancer.

Cox proportional hazards analysis revealed that only gender had a significant impact on overall survival (*p* = 0.019) (Table 3, Figure 3 and Figure 4. BRN was associated with a hazard ratio of 0.229, suggesting a potential trend of longer overall survival compared to recurrent CM. However, this association was not statistically significant (*p* = 0.098) (Table 3).

The log-rank test demonstrated a significant difference in survival rate (*p* = 0.002) between patients with CM and BRN, as shown in the Kaplan–Meier curves (Figure 2 and Figure 3). However, this difference in overall survival became insignificant after adjusting for confounding variables (*p* = 0.657). In the unadjusted survival analysis excluding the WBRT group, the log-rank test did not show any difference in group survival (*p* = 0.211).

## 4. Discussion

In this single-center retrospective study, we analyzed a cohort of 28 patients from an initial 113 cases, focusing on the diagnostic accuracy of conventional MRI and MR perfusion scans in differentiating between recurrent cerebral metastasis (CM) and brain radiation necrosis (BRN). Conventional MRI correctly identified recurrent CM in 75% of cases, while MR perfusion scans identified BRN in 100% of cases. The difference in diagnostic accuracy between conventional MRI and MR perfusion was not statistically significant, however.

MR perfusion imaging showed higher mean values of maximum relative cerebral blood volume in recurrent CM than in BRN, suggesting its potential as a discriminative marker. The mean values of max. rCBF also tended to be higher in recurrent CM, nearly reaching statistical significance. Non-invasive discrimination of the two lesions would have significant clinical implications. At present, the diagnosis is first apparent after invasive treatment, typically surgical resection. If BRN could be reliably identified by imaging, this population of patients would, in many cases, avoid surgical resection, with its associated risk. BRN could in many cases be treated with, for example, corticosteroids. The current diagnostic challenges limit this option, as corticosteroids may mask the progression of CM. The rationale behind the comparison between the two modalities was to assess whether the (additional) MRI perfusion scan provided supplementary diagnostic value, rather than indicating the superiority of one technique over the other. Typically, MRIs are available at the time of initial evaluation. In our center, clinicians have sometimes used MRI perfusion as an additional modality in situations where further diagnostic evaluation is warranted before intervention.

Survival analysis indicated gender as the only variable significantly affecting overall survival. While BRN patients tended to have longer survival than those with recurrent CM, this did not reach statistical significance once other variables were adjusted for. The initial apparent survival difference between CM and BRN patients seen in the log-rank test was negated after adjustments for confounders.

### 4.1. Comparison with Previous Studies

Studies investigating the accuracy of MR perfusion scans have focused on distinguishing primary brain neoplasms (typically high-grade gliomas) from BRN, rather than CM from BRN [13,15,16]. Relative cerebral blood volume is a widely used quantitative parameter [15] and our study also suggested that this was significantly increased in patients with recurrent CM.

Frequently used parameters of interest in conventional MRI scans are the morphological appearance of contrast enhancement, the lesion quotient, the size of the surrounding edema, and T1/T2 mismatch [17,18]. The growing use of immune therapy may increase the uncertainty related to conventional MRI as the immune response tends to mimic the lesion enhancements of CM on conventional MRI scans [10].

Studies to determine the optimal non-invasive technique have shown inconsistent findings [19,20,21], with varying estimated cut-off values for diagnostic parameters [8,17,22,23]. Mitsuya et al. identified a specific rCBV ratio threshold (greater than 2.1) with high sensitivity and specificity for detecting recurrent metastatic tumors, but our findings were less definitive [18]. This discrepancy could be attributed to variations in our study’s retrospective design, patient selection, or imaging parameters. Our results, alongside Mitsuya et al.’s findings, highlight the potential of MR perfusion in neuro-oncological diagnostics, but also underscore the need for larger, perhaps more diverse studies to further validate and refine the use of rCBV ratios in clinical decision-making.

In the current study, we found that neither conventional MRI nor MR perfusion scans could reliably distinguish between CM and BRN. The estimated positive predictive value of raising suspicion of recurrent CM was 75% for conventional MRI and 100% for MR perfusion scan. A meta-analysis of five scan types, including conventional contrast-enhanced MRI and MR perfusion, [22] showed that all modalities exhibited high sensitivity for diagnosing recurrent CM but inadequate specificity, hence often misdiagnosing BRN as CM. The apparent implication is an overselection of patients for invasive treatment (i.e., surgical resection). It may be argued that this is favorable to the opposite situation, as wrongfully diagnosing CM as BRN may delay correct treatment and allow for tumor progression. A retrospective study of conventional MRI [24] to investigate the predictive value of several diagnostic parameters such as the lesion quotient found that all parameters were insufficient to distinguish BRN from CM. Another review [25] concluded that morphological imaging such as conventional contrast-enhanced MRI could be misleading and that MR perfusion scans have limited use in distinguishing BRN from CM due to the complicated quantification of some perfusion parameters and a limited spatial image resolution.

### 4.2. Survival Analysis

After controlling for confounding factors, we found no statistically significant difference in survival between patients with BRN and those with recurrent CM. There was, however, an observed trend in longer overall survival in patients with BRN. This is in agreement with current knowledge, as BRN typically has a better prognosis than CM [9]. The current treatment regime, with extensive use of invasive diagnostics, may be a contributing factor in the observed survival trends. The exclusion of patients with CM unavailable for surgical resection warrants a comment. This group may have had a worse overall prognosis. If these people had been included in the survival analysis, the results may have been shifted further in favor of BRN [26].

Female patients showed longer overall survival, possibly influenced by the prevalence of breast cancer (15%) among the female patients. This factor might also contribute to the higher survival rates observed in the BRN group in the unadjusted analysis. Contrary to expectations, age did not significantly impact survival. This remained true even after accounting for ASA score, imaging modality, and radiation exposure, which were also not predictors of survival. No significant age-based differences were found regarding gender distribution, cancer type, or surgical outcomes. The lack of data on specific oncologic treatments pre- and post-surgery limits further insights into their potential impact on survival.

### 4.3. Study Limitations

The limitations of this study were its retrospective design and relatively small cohort size, and these could have led to residual confounding and type I and II errors. Our study involved 32 cases from 28 patients, which, while providing valuable preliminary insights, is indeed a limited cohort; in particular, the potential for a type II statistical error is increased due to the small sample size. Larger, prospective studies are necessary to validate our findings. The retrospective nature limits the control over oncological treatment variables, which can significantly influence radiological appearances and thus the interpretability of imaging results. Future studies should be prospective and multicenter to provide a more robust dataset and have better control over treatment variables.

Another limitation relates to selection bias in imaging modality choice. Patients with scans indicative of extensive metastatic disease often did not undergo additional MR perfusion scans if the conventional MRI findings were overtly malignant, potentially skewing true CM predictions. Conversely, if CM was suspected in previously irradiated sites, MR perfusion scans were more frequently employed, and this may have led to an overestimation of conventional MRI capabilities and an underestimation of MR perfusion’s diagnostic utility. Dynamic contrast-enhanced (DCE) perfusion-weighted imaging (PWI) represents an alternative and understudied approach for the estimation of pseudo-progression. This approach shows promising results in terms of postoperative evaluation of high-grade gliomas, but the current studies vary widely in their methodology [27].

Furthermore, this study did not account for the impact of various oncological treatments post-initial imaging, which may have altered the radiological landscape and affected the subsequent imaging interpretations. The effects of radiation therapy, chemotherapy, and targeted or immune therapies on brain tissue can complicate the distinction between CM and BRN, introducing additional variables that were not controlled for in this study. This should be addressed in future research.

## 5. Conclusions

This study, involving 28 patients with previous intracranial metastatic cancer, revealed that conventional MRI had a 75% positive predictive value for detecting recurrent cerebral metastasis. MR perfusion showed promise in consistently diagnosing brain radiation necrosis (BRN), particularly by demonstrating higher maximum relative cerebral blood volume (rCBV) in metastatic cases. While our data suggest some potential for MR perfusion in differentiating between recurrent CM and BRN, this differentiation was not definitive across all cases. It is important to note that these observations are derived from a limited patient cohort. These findings underscore the need for large, prospective studies to more accurately assess the diagnostic accuracy of these modalities and to inform treatment decisions in the management of patients with suspected cerebral metastasis.

## Figures and Tables

**Table 1:** Baseline characteristics.

		Contrast MRI, *n* = 24	MR Perfusion, *n* = 8	*p*-Value
Age at initial CM diagnosis, median (IQR)		65 (8)	64 (7)	0.597
Female		12 (50%)	4 (50%)	
Mortality (%)		18 (75%)	4 (50%)	0.060
Preoperative ASA score	1	0	0	0.926
	2	9	4	
	3	14	5	
	4	1	0	
Primary cancer	NSCLC, SCLC	10	4	0.754
	Gastrointestinal	3	0	
	Urogenital	4	2	
	Breast	3	0	
	Malignant melanoma	3	2	
	Other	1	0	
Radiation exposure	WBRT	6	0	0.147
	SRS	13	8	
	Both	5	1	
Months from RT to surgery, median (IQR)		7.5 (8.5)	8 (8)	0.913
≥6 months from RT to surgery (%)		19 (79%)	7 (78%)	
		Radiation modaility	Dose(Gy)/fractions (WBRT:SRS)	Number
		WBRT	30/10	5
		SRS	20/1	5
			27/3	9
			30/5	3
			30/10	1
		Both	30/10:27/3	2
			30/10:27/1	1
			30/10:30/5	1
			30/10:36/13	1

Abbreviations: CM, cerebral metastasis; IQR, interquartile range; NSCLC, non-small lung cell cancer; SCLC, small cell lung cancer; ASA, American Society of Anesthesiologists score; WBRT, whole-brain radiotherapy; SRS, stereotactic radiosurgery; RT, radiotherapy; IQR, interquartile range.

**Table 2:** Diagnostic accuracy and imaging parameters for the eight patients undergoing MR perfusion scans, with histological diagnosis of metastasis or radiation necrosis.

	Histological Metastasis (*n* = 4)	Histological Radiation Necrosis (*n* = 4)	*p*-Value
Consistent radiological diagnosis	3 (75%)	4 (100%)	0.14
Max. rCBV, mean (SD)	2.52 (1.34)	1.14 (0.45)	0.048
Max. rCBF, mean (SD)	1.78 (0.80)	0.96 (0.28)	0.051

Abbreviations: rCBV, relative cerebral blood volume; rCBF, relative cerebral blood flow; SD, standard deviation.

**Table 3:** Cox proportional hazards model for survival.

	HR (95% CI)	*p*-Value
Diagnosis (BRN)	0.229 (0.040–1.310)	0.098
Scan (MR perfusion)	0.779 (0.234–2.584)	0.683
Gender (female)	0.243 (0.075–0.791)	0.019
Age (≥65)	1.415 (0.342–5.839)	0.631
Radiation exposure	1.487 (0.721–3.066)	0.282
ASA score	0.573 (0.198–1.655)	0.304

Abbreviations: BRN, brain radiation necrosis; HR, hazard ratio; ASA, American Society of Anesthesiologists.

## Data Availability

The datasets presented in this article are not readily available because of national legislation. Requests to access the datasets should be directed to frantz.rom.poulsen@rsyd.dk.

## Supplementary Materials

The following supporting information can be downloaded at: https://www.mdpi.com/article/10.3390/brainsci14040321/s1, Supplementary Table S1: Detailed MR perfusion imaging parameters and diagnostic outcomes for individual patients.
