# Supplementary material for: Evaluating the Efficacy of Perfusion MRI and Conventional MRI in Distinguishing Recurrent Cerebral Metastasis from Brain Radiation Necrosis
Source: Brain Sci. 2024 Mar 27;14(4):321. doi: 10.3390/brainsci14040321 (PMC11048647; doi:10.3390/brainsci14040321)
Supplement: Supplementary file 1 [file brainsci-14-00321-s001.zip › brainsci-2915821-supplementary.pdf]

Supplementary Table S1: Detailed MR perfusion imaging parameters and diagnostic outcomes for individual patients

This table presents individual patient data from MR perfusion imaging, including the relative cerebral blood volume (rCBV), maximum rCBV (Max rCBV), relative cerebral blood flow (rCBF) index, and maximum rCBF (Max rCBF). It also documents the initial radiological diagnosis compared to the final histological diagnosis for each of the eight patients included in the study. Abbreviation: rad: Brain radiation necrosis, met: recurrent cerebral metastasis.

id	rCBV	Max rCBV	rCBF	Max rCBF	Radiological diagnosis	Histological diagnosis
1	0.03692327	0.97	0.03	0.95	rad	met
	0.03666318		0.04			
	0.42935189		0.95			
	0.36673445		0.41			
	0.96915814		0.34			
2	0.30051924	3.09	0.28	2.73	met	met
	1.29705762		1.18			
	3.09246601		2.73			
	0.7810319		1.03			
	0.48013591		0.51			
3	1.38815604	1.66	1.17	1.26	rad	rad
	0.5725037		0.59			
	1.10699567		1.05			
	1.52870334		1.26			
	1.66257968		1.20			
4	0.35413797	0.58	0.36	0.59	rad	rad
	0.54291831		0.55			
	0.58015713		0.59			
5	1.27065303	1.27	0.96	0.96	rad	rad
	0.86159625		0.72			
	0.79338747		0.62			
	0.82720421		0.80			
6	1.99175932	1.99	1.30	1.30	met	met

	0.97369451		0.95			
	1.28701084		1.06			
	0.90160929		0.78			
	1.85245699		1.23			
7	1.02161153	1.04	1.02	1.03	rad	rad
	1.04376987		1.03			
	0.91534415		0.91			
8	4.05028015	4.05	2.12	2.12	met	met
	1.90404483		1.30			
	1.43945838		0.99			
	1.54972961		1.27			
